# O11.3. A LONGITUDINAL ANALYSIS OF THE EFFECTS OF NEUROTICISM AND EXTRAVERSION ON SUBJECTIVE WELL-BEING IN PATIENTS WITH SCHIZOPHRENIA

**DOI:** 10.1093/schbul/sby015.263

**Published:** 2018-04-01

**Authors:** Floor van Dijk, Frederike Schirmbeck, Lieuwe de Haan

**Affiliations:** Academic Medical Centre, University of Amsterdam

## Abstract

**Background:**

One in five patients with a psychotic disorder has a persistent low subjective well-being over three years. This group has a poorer prognosis for social functioning. This presentation reports on the first longitudinal study evaluating whether neuroticism and extraversion influence subjective well-being (SWB) in patients with a schizophrenia spectrum disorder. SWB is generally defined as ‘the subjective experience, as constituting aspects of mental or physical state, which patients report regardless of etiological attributions’. It is an independent determinant for recovery in patients with a psychotic disorder. Two cross-sectional studies on quality of life in schizophrenia suggest that personality traits are associated with the way patients value life. If personality traits predict the trajectories of subjective well-being, our results would provide a clinical reference point for patients at risk for a persistent low subjective well-being.

**Methods:**

We included 186 patients and 126 healthy control subjects from the Dutch Genetic Risk and Risk and Outcome of Psychosis cohort. SWB was measured with the Subjective Well-being under Neuroleptics-20 (SWN) scale. Assessments took place at baseline, three years and six years follow-up. We used the Five-Factor Inventory to assess neuroticism and extraversion. Positive, negative and depressive symptoms in patients were assessed by the Positive and Negative Symptoms Scale. For controls we used the Community Assessment of Psychic Experiences for investigating subclinical symptoms. By using linear mixed model analyses we investigated the relation between SWB and the personality traits, including the moderating associations of positive, negative, depressive symptoms and a range of psychosocial indicators (among which antipsychotic use and smoking cannabis). An exploratory analysis in the patient sample, investigated the predictive values of personality traits and symptoms at baseline on the course of SWB over 3 and 6 years. Patients were accounted to one of three SWB-trajectories ‘stable low’, ‘low start and improving’ and ‘stable high’.

**Results:**

Mixed model analyses revealed that in patients, high scores of neuroticism and low scores of extraversion were associated with lower SWN-scores: at 3 years: t = - 3.07 and t = 4.34 for p < 0.05 and at 6 years: t = -2.62, p = 0.009 and t = 3.51, p = 0.001. We found no interaction effect of time and personality traits. Neuroticism and extraversion were related to SWB to the same extent in the control group.

Regarding trajectories over time, we found a stable low SWB in 15.1% of the patients, forming the ‘stable low’ trajectory group. This group scored highest on neuroticism and lowest on extraversion compared to patients with an increase in SWB or a stable high SWB: neuroticism scores showed post hoc compared mean differences (MD) of 4.25, p = 0.03 for the ‘low start increasing’-group and MD 10.75, p <0.001 for the ‘stable high’-group).

**Discussion:**

We found an association between personality traits and subjective well-being regardless of (subclinical) psychotic or depressive symptoms. Extraversion can be regarded as a resilience factor, whereas neuroticism is associated with a persistent low well-being. In patient with a schizophrenia spectrum disorder, neuroticism could be a focus for therapeutic interventions that diminish negative affectivity. Additionally, an assessment of neuroticism and extraversion early in the process of treatment could be considered.

